# Childhood Laryngeal Dystonia Following Bilateral Globus Pallidus Abnormality: A Case Study and Review of Literature

**Published:** 2017-01

**Authors:** Mohammad Javad Saeedi Borujeni, Ebrahim Esfandiary, Mostafa Almasi-Dooghaee

**Affiliations:** 1 *Department ofAnatomical Sciences and Molecular Biology, School of Medicine, Isfahan University of Medical Sciences, Isfahan, Iran.*; 2 *Department of Neurology, Iran University of Medical Sciences, Tehran, Iran.*

**Keywords:** Basal ganglia, Laryngeal dystonia, Globus pallidus

## Abstract

**Introduction:**

Dystonia is a disorder of movement caused by various etiologies. Laryngeal dystonia is caused by the spasm of laryngeal muscles. It is a disorder caused by vocal fold movement in which excessive adduction or abduction of the vocal folds occurs during speech. The pathophysiology of this type of dystonia is not fully known. Some researchers have suggested that basal ganglia structures and their connections with cortical areas have been involved in the pathogenesis of dystonia.

**Case Report:**

In this paper a 7.5-year-old boy suffering from laryngeal dystonia with bilateral lesions in Globus Pallidus is presented. The patient also suffered from swallowing problems, monotone voice, vocal tremor, hypersensitivity of gag reflex, and stuttering. Drug treatment failed to cure him; therefore, he was referred to rehabilitation therapy.

**Conclusion:**

In conclusion, special attention should be brought upon laryngeal dystonia, especially in patients showing Extra-pyramidal symptoms and/or abnormalities of the basal ganglia. In children, laryngeal dystonia may be potentially fatal. Lack of consideration for this condition during rehabilitation therapy can lead to serious consequences for a child.

## Introduction

One of the potentially fatal types of dystonia is laryngeal dystonia (LD), which is characterized by laryngeal stridor following dysfunction of the vocal fold. LD can occur due to the involvement of the extrapyramidal system in neurodege- nerative diseases (1,2). LD can be threatening to life because of the risk and danger of respiratory blocks (3). LD is a movement disorder of the intrinsic laryngeal muscles, which most generally manifests as spasmodic dysphonia (SD) (1). SD is an infrequent speech disorder characterized by breaks in the voice and an obvious effort during speaking (4). SD is the 3^rd ^most common form of dystonia and its estimated prevalence is 1 per 100,000 (5).

The basal ganglia are the largest subcortical structures in the human brain. They are placed in an essential position, which influences motor and emotional behaviors (6). The main components of the basal ganglia are the Caudate nucleus, Lentiform nucleus, substantia nigra, the subthalamic nucleus, and Claustrum. The lentiform nucleus lies lateral to the internal capsule and is divided into a lateral part (the putamen) and medial part (the Globus Pallidus) (GP). The GP is further subdivided into internal and external segments. GP is one of the main components of the motor circuit of the basal ganglia and affects the cerebral cortex output via connections with the putamen, subthalamic nucleus, and thalamus (direct and indirect motor circuit) (6). 

Bilateral signal change in the basal ganglia in a MRI has some differential diagnosis including toxic poisoning (e.g. carbon monoxide, methanol, and cyanide), metabolic disorders (e.g. acquired hepatocerebral degeneration, Non- ketotic hyperglycemia, hypoglycemia, osmotic myelinolysis, Wernicke encephalopathy), hypoxic ischemic encephalopathy, mithocondrial disorders (especially Leigh disease), neurodegenerative disorders (e.g.Wilson disease, Neurodegeneration with brain iron accumu- lation), Creutzfeldt-Jakob disease, Fahr disease, vascular events involving basal ganglia (deep cerebral venous thrombosis, basilar artery occlusion, Behçet disease), and some neoplastic disorders (e.g. primary bilateral thalamic glioma, primary CNS lymphoma). Many of these differential diagnoses involve other parts of the brain in addition to the basal ganglia; for example, hypoxia, hypoglycemia may involve the cerebral cortex; and poisoning, hypoglyce- mia and primary CNS lymphoma may involve cerebral white matter. The brainstem lesions may be seen in Wernicke encephalo- pathy, Leigh disease, osmotic myelinolysis, Behçet disease, and basilar artery occlusion. In addition, the appearance of the lesion in a CT scan and different sequences of MRI including T1 and T2-weighted imaging and also contrast enhancement with gadolinium can help in the diagnosis of the disease (7,8).

Some researchers have suggested that basal ganglia structures and their cortical connections have been implicated in the pathogenesis of dystonia (9). In addition, LD has been reported following lesions in the cerebellum and sensorimotor cortex (10-12).

This paper examines the report of congenital LD due to bilateral basal ganglia abnormality in globus pallidus, and is followed by a description and investigation of the possible causes of this type of dystonia. 

## Case Report

A 7.5–year-old boy was referred with multiple problems such as balance disturbances, incoordination of extra ocular muscles during walking, disorders of gag reflex and swallowing, vocal tremor, monotone speech, and weakness of mastication muscles. History of delayed motor development was positive. One of the important and threatening problems in the patient was LD. There was no LD in the patient’s family history. The patient’s grandfather suffered from Parkinson’s disease in his late life. There was no history of delayed delivery or cyanosis after birth. The visiting psychologist did not report any mental disabilities in the child. 

The routine laboratory tests including serum electrolytes, blood glucose level, liver function tests, and kidney function tests were normal. In the urine and blood chromatography tests, there were no bands in the urine and no detectable increase in any particular amino acid in the plasma sample, in favor of any inherited or acquired metabolic disorders. Level of plasma Copper was in the normal range (90 Mic g /dl) and there was no evidence of mitochondrial disorders. The plasma lactate level was in normal range (9.6 mg/dl).

**Fig1:**
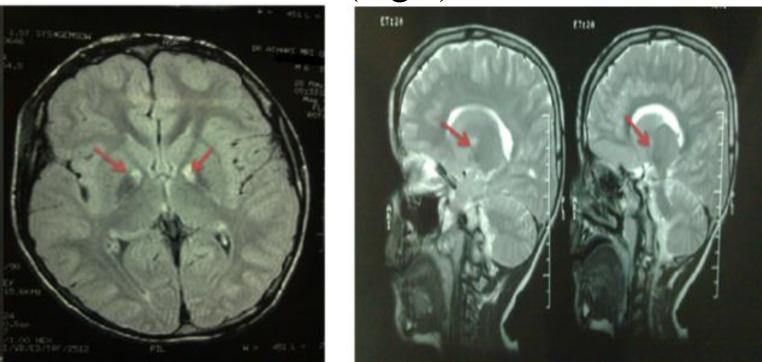
Magnetic resonance imaging (MRI) scans of patient showing bilateral Globus pallidus (GP) abnormality (red arrow). **Lef**t: Axial FLAIR brain MRI, shows hyposignality in bilateral GP with a hypersignality in its medial part; **Right**: Sagittal T2-weighted brain MRI shows hypersignality in medial part of GP.

The patient was aware and exhibited normal behaviors. During the language skills assessment, he had normal understanding, naming, and repetition. His cranial nerve examination revealed that the pupil response to light and accommodation was normal. Fields of vision were full to confrontation testing. There were no abnormalities during the funduscopic test. The Kayser–Fleischer ring did not appear around the corneo-scleral junction. Incoordination in the extra ocular muscles was shown during walking. Sense of facial region was normal to prick and touch. The face moved symmetrically. Soft palate and uvula rose in the midline and the tongue protrusion was in the midline. Deep tendon reflexes were exaggerated with a pattern suggesting upper motor neuron disease. He had bilateral babinsky sign. Among neonatal reflexes, the biting reflex remained. Cerebellar examination was normal. The masticatory muscles were weak. Hypersensitivity of the gag reflex was present. Results of a magnetic resonance imaging (MRI) brain scan demonstrated bilateral basal ganglia abnormality in the Globus Pallidus (Fig.1). 

The ventricular system, aqueduct, C.S.F, and brain parenchyma were normal. No tumor, signal change, or midline shift was noted. The pons, brachium cerebelli, cerebello- pontine angles, parasellar, perisellar, sellar regions and pituitary gland were normal. The medulla oblongata, bulb and upper cervical cord showed no abnormalities.

The results of computerized tomography (CT) scans confirmed that the density of the white and gray matter was within normal limits. The BAEPs, SEPs, Flash-VEPs types of evoked potential^’^s report (EP) indicated that the morphology of the waves was normal. In addition, it was observed that wave’s amplitudes and latencies were normal. The voice is a multidimensional process. Audio recording is the most important basic requisite for the assessment of voice. For this purpose, we recorded the patient speaking for 10 minutes using the jet audio software (8.1.3.2200 Plus version). Upon listening to the speech sample, based on the auditory perceptual assessment, we diagnosed some items: (A) Breathiness of voice: the patient’s voice was breathy which can be a sign of air leakage through an insufficient glottis closure. (B) Harshness of voice: our auditory perception showed that the patient’s voice was rough. This condition can be a sign of irregular glottis pulses. (C) Vocal tremor: tremor of voice was perceptible. (D) Hyper nasality of voice: In our opinion, there was no rhinophonia or excessive resonance of voice in the nasal cavity and paranasal sinuses. (E) Pitch of voice: the patient’s voice was roughly monotone in pitch. (F) Breathy breaks: this condition was recognized in the speech profile. To conduct a phonation airflow examination, the simplest method for the assessment of the aerodynamic parameter of the voice is the maximum phonation timing (MPT) (in seconds). It consists of the prolongation of a /a: /for as long as possible after maximum inspiration.The average time of MPT in our patient was 5 seconds.

Because of the lack of The Kayser–Fleischer ring and the normal range of copper plasma level, Wilson’s disease was not considered as a diagnosis for this patient. According to the differential diagnoses list mentioned earlier, neurodegeneration with brain iron accumulation (NBIA) especially Hallervorden-Spatz syndrome could be a possible diagnosis for this patient. This requires a genetic study for the detection of the PANK-2 gene (13); however, his genetic study findings were not available. It seems that this patient has a congenital abnormality in the Globus Pallidus, which is caused by an unclear reason. Overall, when compared to the criteria usually accepted to describe abductor LD (14), the patient’s speech profile did conform many of those characteristics. 

After a 3-month treatment period using voice rehabilitation methods such as: laryngeal manipulation, vocal relaxation, respiratory exercise and mental aspects, the patient’s symptoms improved.

## Discussion

LD includes vocal folds dystonia during phonation and respiration. The two main types of LD have been identified: adductor LD, in which there is an abnormal hyper adduction of the vocal cords during speaking creating a strain-strangled voice; and abductor LD, in which there is unsuitable abductor spasms when speaking, which leads to the production of breathy breaks (11). LD symptoms may decrease during sleep and can be increased by tension and emotion. Disorders of the extra–pyramidal system, such as Parkinson’s disease, are often complicated by LD (15). In our patient, the disturbance of respiration completely disappeared during sleep and was increased by tension or emotion. He presented extra-pyramidal lesion symptoms, which were confirmed basal ganglia anomalies detected during para clinical diagnosis. Some of the risk factors for LD are being a young males, having a positive history of hypersensitivity, and basal ganglia anomalies (16). In this patient, hypersensitivity of gag reflex was present and basal ganglia lesions were reported in an MRI scan. Investigations on the neurophysiological basis of dystonia have shown that sensory anomalies are present in parts of the body that may or may not be affected by dystonia (17,18). The sensory abnormality present in our patient was hypersensitivity.

One of the strongest support for the involvement of basal ganglia in LD originates from pharmacological research. It seems that abnormal nigrostriatal dopamine activity is associated with the pathological basis of extra-pyramidal adverse response (3). Russel et al. (1996) showed that any treatment which antagonizes nigrostriatal dopamine function can produce dystonia (19). Miyata et al.’s (2010) survey also recommended that levodopa in a low dose be used for treatment of uncontrolled movement in LD patients (20).This patient referred to the speech therapy facility of the hospital with multiple problems, in addition to LD. Literature review has made it clear that a definitive diagnosis of LD type is difficult. It seems that our patient has some degree of abductor LD. For a definitive diagnosis of LD, laryngeal stroboscopic test is useful; however, because of special conditions associated with the patient and the opposing view of his physician, this test was not conducted.

## Conclusion

In conclusion, LD should be taken into consideration, especially in patients showing Extra-Pyramidal symptoms and/or abnormalities of basal ganglia. In children, LD may be potentially fatal. Lack of consideration for this condition during rehabilitation therapy can lead to serious consequences in children.
